# Cognitive Offloading Does Not Prevent but Rather Promotes Cognitive Development

**DOI:** 10.1371/journal.pone.0160679

**Published:** 2016-08-09

**Authors:** Jônata Tyska Carvalho, Stefano Nolfi

**Affiliations:** 1 Institute of Cognitive Sciences and Technologies, National Research Council (CNR), Via S. Martino della Battaglia, 44, 00185, Roma, Italia; 2 Center for Computational Sciences (C3), Federal University of Rio Grande (FURG), Av. Italia, km 8, 96203–900, Rio Grande, Brasil; Nathan S Kline Institute, UNITED STATES

## Abstract

We investigate the relation between the development of reactive and cognitive capabilities. In particular we investigate whether the development of reactive capabilities prevents or promotes the development of cognitive capabilities in a population of evolving robots that have to solve a time-delay navigation task in a double T-Maze environment. Analysis of the experiments reveals that the evolving robots always select reactive strategies that rely on cognitive offloading, i.e., the possibility of acting so as to encode onto the relation between the agent and the environment the states that can be used later to regulate the agent’s behavior. The discovery of these strategies does not prevent, but rather facilitates, the development of cognitive strategies that also rely on the extraction and use of internal states. Detailed analysis of the results obtained in the different experimental conditions provides evidence that helps clarify why, contrary to expectations, reactive and cognitive strategies tend to have synergetic relationships.

## Introduction

Developments in psychology, neuroscience, linguistics, robotics and philosophy have clarified that cognition cannot be studied properly without taking into sufficient account the role of the body, action and the external world [1–7]. The agent’s body and the environment in which it is situated provide a great deal of structure that is used to operate appropriately. Consequently, in many cases the internal capabilities required are much simpler than those previously hypothesized within disembodied accounts. For example, moving around in a city does not necessarily require an elaborate representation of the city’s layout. The ability to recognize a limited number of turning decision points combined with the ability to just follow the street between decision points might suffice [8]. Similarly, baseball players do not need to estimate the trajectory of the flying ball to be intercepted through complex calculations. They can simply adjust their running speed so as to maintain the relative angle between their eyes and the ball constant [9].

Exploitation of the information that can be extracted directly from the environment and of the effects of situated actions do not only affect the agents’ low-level capabilities. Embodied and embedded strategies (like those described above) co-exist and interact with different strategies that are less dependent on agent/environmental interactions and more dependent on internal processes at all levels of organization [5]. However, the relation and the interaction between strategies and capabilities that differ in that respect have not yet been investigated. Consequently, the question of how these different types of strategies can be integrated from an operational and developmental perspective is still open. In particular, one important question that needs to be answered is the following: “Is cognition truly seamless–implying a gentle, incremental trajectory linking fully embodied responsiveness to abstract thought and off-line reason? Or is it a patchwork quilt, with jumps and discontinuities and with very different kinds of processing and representations serving different needs?” [5].

In this paper we investigate the relation between the development of reactive and cognitive capabilities. In particular we investigate whether the development of reactive capabilities prevents or promotes the development of cognitive capabilities. For the scope of this paper we define cognition as the ability to integrate sensory-motor information over time into internal states and to use these internal states to regulate the way the agent reacts to perceived stimuli. The term cognition is often used in a more restricted way. In the above definition, we focus on a fundamental capacity that is at the basis of all cognitive capabilities (e.g. perception, memory, attention, decision-making, reasoning, language, etc.).

Evolutionary Robotics [10] is a suitable method for studying the relation between reactive and cognitive abilities in adaptive agents. Indeed research carried out in this area has demonstrated how evolving robots can master both problems that have reactive solutions and problems that require the development of cognitive abilities (see for example [11–19]). However, what has not been sufficiently investigated to date is the relation between reactive and cognitive strategies.

Any adaptive problem typically admits qualitatively different sub-optimal and optimal solutions. Depending on the circumstances, the discovery of one type of sub-optimal solution might facilitate or block the discovery of better alternative solutions. Indeed, the discovery of sub-optimal solutions that cannot be progressively transformed into better solutions without loss of performance should retard or block the discovery of effective solutions. On the contrary, the selection of sub-optimal solutions that can be transformed into better solutions without causing significant performance loss can facilitate the discovery of effective solutions. In the latter case, the strength of the facilitation effect would depend on the level of overlap or similarity between the first and the second solutions.

Since reactive solutions are typically simpler than cognitive solutions from the point of view of the complexity of the required control mechanisms, and since adaptation tends to find the simpler solution first, the question is the following: Can reactive solutions be transformed into better solutions that also include the utilization of internal states without causing significant performance loss and what is the level of overlap/similarity between sub-optimal reactive solutions and better solutions that include cognitive capabilities.

According to some authors, the development of reactive solutions blocks the development of cognitive capabilities. In particular, [20] claimed that reactive solutions constitute hard to escape local optima that prevent the development of cognitive solutions. Similarly, [21] claimed that the deception caused by the availability of locally optimal reactive policies is one of the main factors that explains why it is difficult for cognitive policies to evolve. For these reasons the authors hypothesized that the development of cognitive solutions necessarily requires specific selective cognitive pressures such as fitness components that encourage the development of short-term memory [20] or mechanisms for avoiding local optima, such as novelty search [21].

One aspect that is particularly relevant from the viewpoint of the relation between reactive and cognitive strategies is cognitive offloading, i.e., the possibility of offloading cognitive work onto the environment [22–24]. In particular, the possibility of acting so as to encode the states that can be used to regulate the agent’s behavior onto the external environment and/or onto the relation between the agent and the environment. In fact, the possibility of encoding the required states internally or externally suggests that cognitive strategies and reactive strategies (that rely on cognitive offloading) represent two alternative but functionally equivalent modalities. A simple example of cognitive offloading related to everyday human life is crossing two fingers so to avoid forgetting to perform a certain action [24–26]. An example of cognitive offloading realized in a robotic scenario consists of dropping markers in the environment that are used to find the way back to the home location [19].

Cognitive offloading is usually considered in the case of cognitive agents that already possess cognitive abilities but offload information into the environment or into their relation with the environment. In this work, instead, we focus on a developmental perspective in which the agents need to develop a certain skill to adapt to their environment and can do so by using a reactive strategy that relies on cognitive offloading, a cognitive strategy that relies on internal states, or on a hybrid strategy that relies on both.

In this paper we report a series of experiments in which a population of robots provided with neural network controllers were evolved for the ability to master a navigation problem in a double T-maze environment that required the exhibition of delayed response behavior. Analysis of the experiments reveals that the evolving robots always selected reactive strategies that relied on cognitive offloading. The discovery of these strategies did not prevent but rather facilitated the development of improved strategies that also relied on the extraction and use of internal states. A detailed analysis of the results obtained in different experimental conditions provides evidence that helps clarify why, contrary expectations, reactive and cognitive strategies tend to have synergetic relationships.

## Method

**Fig 1:**
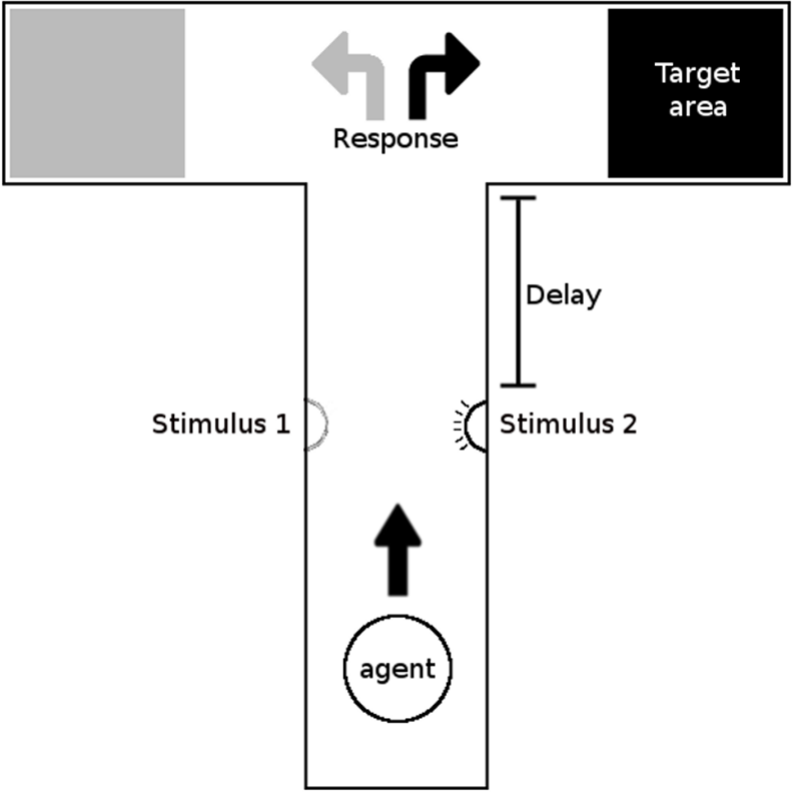
The T-Maze task. The bottom portion of the central corridor includes a stimulus located on the left or the right side. The position of the stimulus corresponds to the position of the target destination. Adapted from [29].

A paradigmatic class of problems that require cognitive skills is constituted by delayed response tasks in which an agent has to act at a certain time t in a conditional dependent manner on the basis of stimuli it encountered at a previous time (t—delay). A simple example of a delayed response task is the so-called road sign task in which an agent that is initially located at the bottom of a T-Maze environment needs to travel toward the top-left or top-right destination by turning left or right at the T-junction depending on whether it previously experienced a stimulus on the left or on the right side of the central corridor, respectively (Fig 1). Therefore, this task was chosen by several researchers to study the evolution of cognitive robots [20, 27–31].

In reality, as demonstrated by [15] this problem has a non-cognitive solution, i.e., a reactive strategy in which the robot always acts on the basis of its current sensory state. Indeed, the robot could solve the task by approaching the experienced stimulus, when visible, and then by following the nearby left or right wall. The availability of simple and optimal reactive solutions of this type prevents the development of cognitive solutions. These reactive solutions, in fact, are easy to discover, optimal and, consequently, evolutionarily stable.

The question that remains open is whether cognitive solutions can evolve in experimental settings that do not allow for optimal reactive solutions or whether the discovery of sub-optimal reactive solutions prevents the discovery of better solutions [20–21] and consequently might drive the evolutionary process toward inescapable local optima. To investigate this question we decided to carry out the evolutionary experiments described in the following sub-sections.

### The robot, the environment, and the task

**Fig 2:**
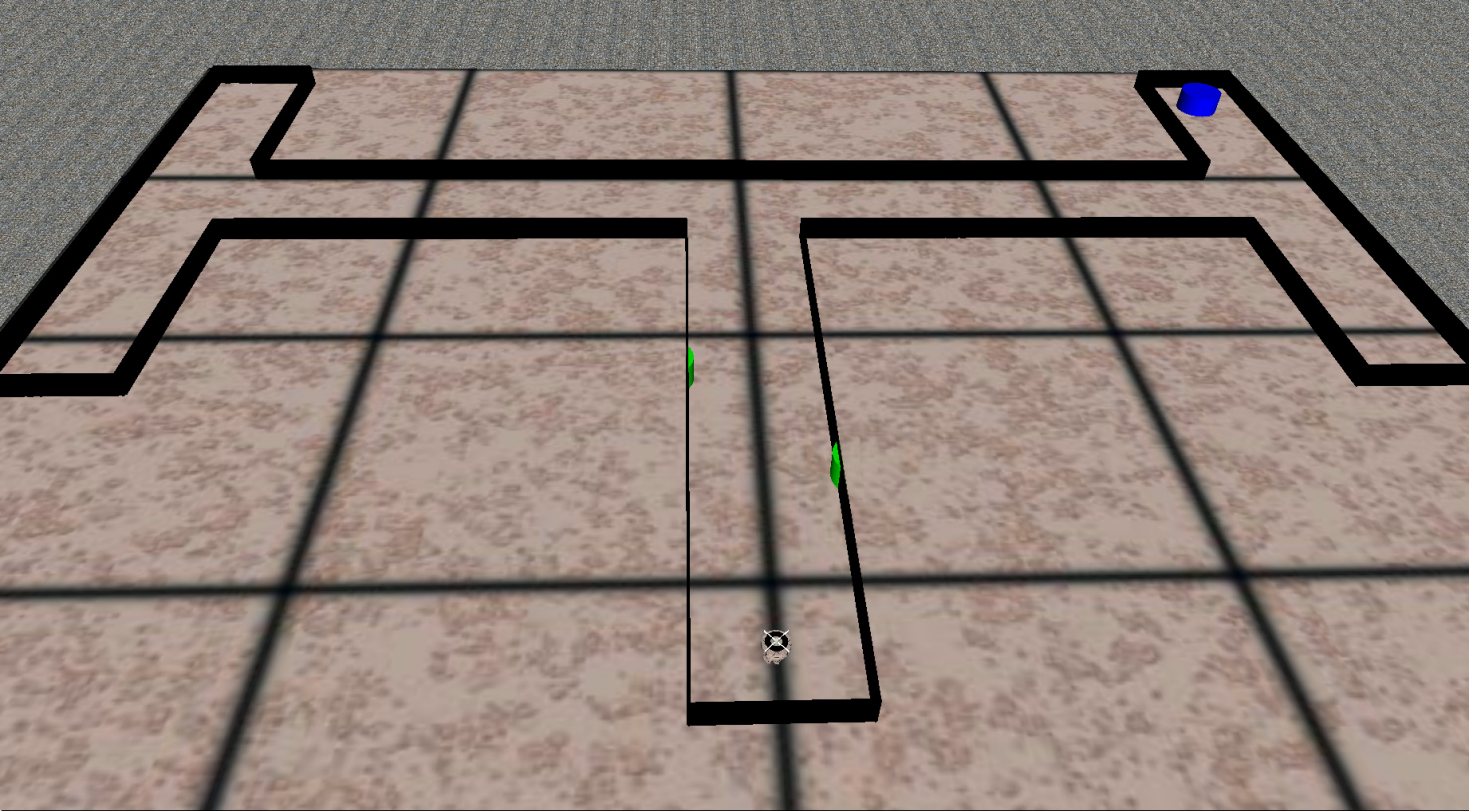
The double T-Maze task. The blue cylinder, which represented the target destination, was located at one of the four end points of the lateral corridors. The central corridor included two green stimuli located in the first and the second part of the corridor on the left or the right side. The position of the first and the second stimulus indicated whether the robot should turn left or right at the first and the second junction, respectively, to reach the target destination.

The environment consisted of a double T-Maze (Fig 2) that included four different destinations and two types of stimuli that could be experienced in four different corresponding patterns (left-left, left-right, right-left, right-right). The increase in complexity with respect to the simple T-Maze environment was due to the fact that the number of destinations to be reached was higher, the number of stimuli experienced was higher, the number of stimuli-dependent decisions that had to be made was higher and the time delay between the moment in which the stimuli were experienced and the moment in which the stimuli-dependent decisions had to be made was longer.

To obtain solutions that were robust with respect to environmental variations, the initial positions and orientations of the robot and the size of the environment were randomly varied at the beginning of each trial. More precisely the length of the central corridor and the two vertical corridors was randomly set during each trial within 4.5m±0.5m and 5.5m±0.5m, respectively. The position between the two signals was also varied proportionally with the length of the central corridor. The initial position of the robot was selected randomly within a 50x45cm rectangular area located at the beginning of the central corridor. The initial orientation of the robot was selected randomly with a uniform distribution in the [-180, 180]° range. Robots were evaluated for 16 or 32 trials, as explained below. Trials lasted up to 1 minute and were stopped as soon as the robot turned in the wrong direction at one of the two junctions.

Complex T-Mazes have already been used in previous research. In particular [32] evolved robots for the ability to navigate in T-Maze environments in multiple trials in which the destination location remained stable. The authors manage to develop robots that were able to “remember” the target location in simple T-Maze but not in double T-Maze environments. [33] evolved robots for the ability to navigate in a triple T-Maze toward 8 alternative destinations. In these experiments, however, the robots were not required to master a delayed response task. Indeed, they received and had access to one of eight different corresponding patterns for the entire duration of each trial.

We used the MarXbot [34] agent, which is a circular robot with a diameter of 17cm equipped with 24 infrared sensors, a rotating scanner sensor and an omnidirectional camera. The experiments were run in simulation by using the FARSA open-software tool that includes an accurate simulator of the robot and of the environment [35]. FARSA has been used to successfully transfer results obtained in simulation to hardware in similar experimental settings (e.g., [17, 36]).

### The robots’ controllers

Evolving robots are provided with neural network controllers. The sensory layer includes eight sensory neurons that encode the average activation state of eight groups of three adjacent infrared sensors, 6 neurons that encode the average activation of the rotating scanner over 60 degrees, and 8 neurons that encode the percentage of green and blue pixels detected in four 90° sectors of the visual field of the camera. The state of the sensory and motor neurons is normalized in the range [0.0, 1.0] and noise is simulated by the addition of random values selected with a uniform distribution in the range [-0.05, 0.05]. The motor layer includes two motor neurons that encode the desired speed of the two corresponding motors (normalized in the range [-10.0,10.0]cm/s) that actuate the differential driving system of the robot.

The experiments were replicated in three different experimental conditions that varied with respect to the architecture of the robots’ neural controller as described below:

(S) Simple: The robots were provided with a simple reactive neural network (that always responded in the same way to the same sensory state) in which the sensory neurons were directly connected to the motor neurons.
(C) Continuous: As in the case of the previous condition the neural network controller included direct sensory-motor connections. In addition, the network included an internal layer with 6 neurons that received connections from the sensory neurons and projected connections to the motor neurons. The internal neurons were continuous, i.e., their output state depended on both the activation received from the incoming connections and on their previous activation state (see [37–38]).
(CR) Continuous Recurrent: The neural network was identical to the previous condition, but the internal neurons were also interconnected through recurrent connections.

The state of the sensors, the neurons and the desired speed of the motors were updated every 50ms. The architecture of the neural network controller was kept fixed.

These experimental conditions were chosen to enable us to verify whether and to what extent the problem had reactive solutions, whether the possibility of integrating sensory-motor information over time into internal neuron states would enable the robots to develop better solutions and whether reactive strategies could coexist with cognitive strategies.

### The evolutionary process

The initial population consisted of 20 randomly generated genotypes that encoded the connection weights, biases, and time constants of the neural network controllers of 20 corresponding individual robots. Each parameter was encoded with 8 bits and normalized in the range [–5.0, +5.0] in the case of connection weights and biases and [0.0, 1.0] in the case of time constants of continuous neurons.

Each individual was allowed to generate one offspring, i.e., a copy of the parent genotype in which each bit was mutated with a given probability. Each offspring was evaluated and was used to replace the genotype of the worst parent or was discarded depending on whether or not it outperformed the worst parent.

Each experiment was repeated 10 times by starting with different randomly initialized genotypes. The evolutionary process continued in two consecutive phases of 2,000 generations. During the first 1,000 generations, the mutation rate was set at 2% and the evolving robots were evaluated in 16 trials. From generation 1,001 on the mutation rate was set at 1%. From generation 1501 to 2000 on the individuals were evaluated in 32 trials. In some of the experiments the robots were subjected to position and orientation noise during the second phase, as described below.

The fitness of the individuals was computed by averaging the fitness obtained during the different trials. The fitness of each trial corresponded to the inverse of the distance, within the maze, between the robot and the target destination at the end of the trial. In other words, the robots were rewarded for the ability to approach the appropriate destinations.

The experiments described in this paper can be replicated by downloading and installing FARSA and the associated experimental plugin from “https://sourceforge.net/projects/farsa/” and from “http://laral.istc.cnr.it/cognoffpone/dtmaze.tgz”.

## Results

In this section we report the results obtained in the different experimental conditions described in Section 2.2 and in additional control experiments described below that were carried out to clarify the role of cognitive offloading in the development of cognitive skills.

**Fig 3:**
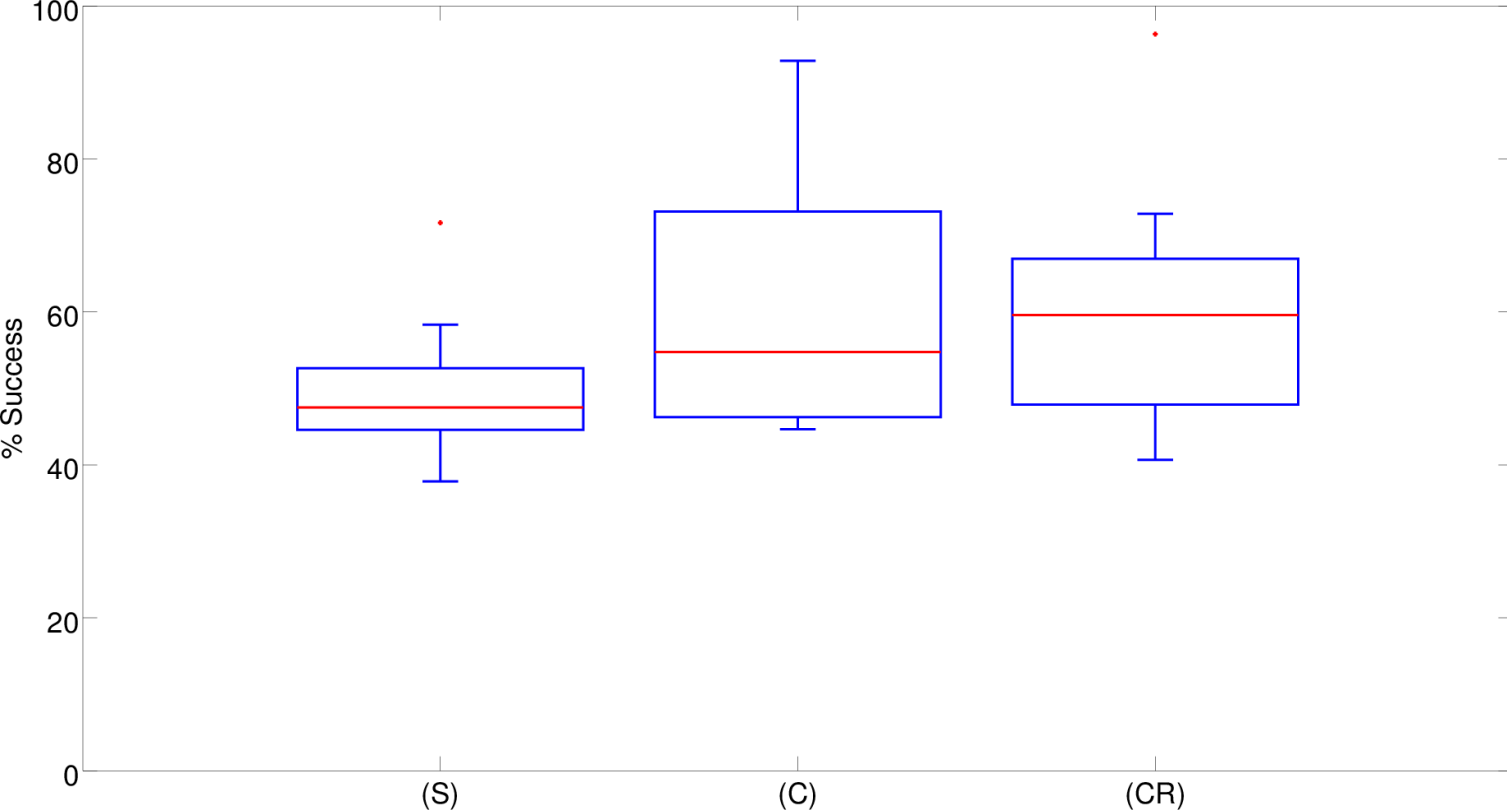
Performance (i.e. percentage of successful trials) of the best 10 robots evolved in the (S), (C) and (CR) experimental conditions in 10 corresponding replications of each experiment. Boxes represent the inter-quartile range of the data and horizontal lines inside the boxes mark the median values. The whiskers extend to the most extreme data points within 1.5 times the inter-quartile range from the box. Circles indicate the outliers.

Overall the performance of the robots (i.e. the percentage of trials in which the evolved robots reached the correct target destination) did not differ significantly between the three experimental conditions in which they were provided with different neural controllers (see Fig 3). By analyzing the performance of the best robot obtained in each experimental condition (Fig 3) we can see how the best (C) and (CR) robots managed to achieve close to optimal performance in 92.8% and 96.3% of the trials, respectively, while the best (S) robot only achieved sub-optimal performance (i.e. 71.7% success). The performance of the best (C) and (CR) robots did not differ significantly (Fisher Exact Test, p = 0.308). The performance of the (S) robot, instead, was significantly worse than the performance of the (C) and (CR) robots (Fisher Exact Test, p<0.001). The data were obtained by post-evaluating the best robot from the last generation of each replication in 600 trials.

These results confirm that, at least in our experimental setup, the double T-Maze problem cannot be solved through simple reactive solutions, although the best reactive robot (S) achieved a remarkably high performance (i.e. by navigating toward the correct destination in 71.7% of cases). Moreover, these results indicate that the possibility of integrating sensory-motor information over time through continuous neurons (C) and recurrent connections (CR) into internal states that are used to regulate the way the robots react to sensory stimuli enables the evolving robots to achieve close-to-optimal performances. On the other hand, the fact that close to optimal performances were achieved in a minority of the replications indicates that the task is hard and there is a high probability that evolution remains stuck in sub-optimal regions of the search space.

**Fig 4:**
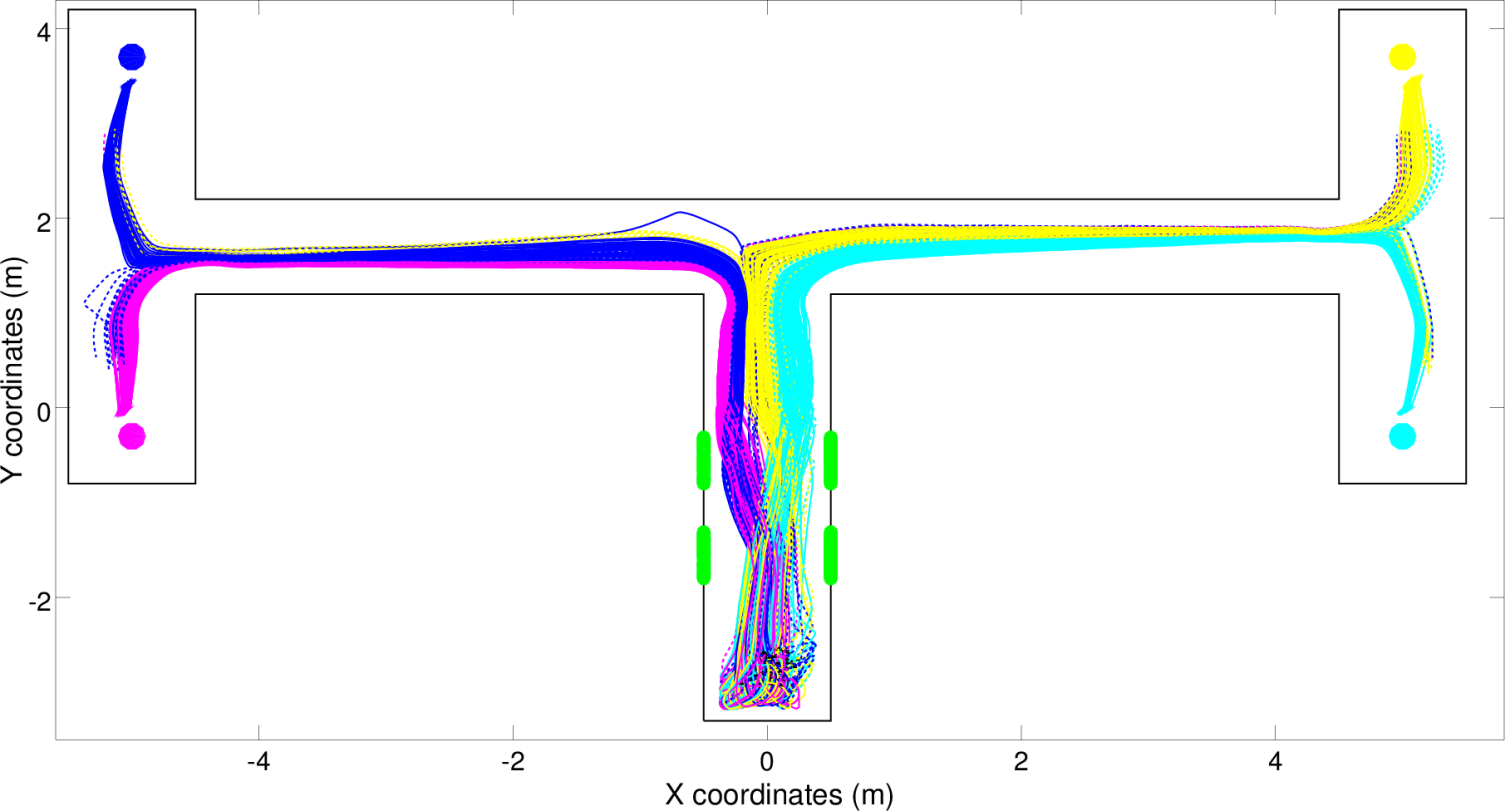
Trajectories produced by the best (S) robot over 300 trials. Full and dashed lines indicate successful and unsuccessful trials, respectively. The color indicates the corresponding target destination (magenta: left-bottom, blue: left-top, cyan: right-bottom, and yellow: right-top).

By inspecting the trajectories produced by the best (S) robot, i.e., by means of a simple reactive controller, we can see how the robot navigated correctly toward all four target destinations most of the time, but erroneously navigated toward the left-left and right-left destinations instead of the left-right destination in several trials (Fig 4, blue trajectories). Surprisingly, this shows that the double T-Maze task can also be solved to a large extent with a reactive solution in which the robot’s actions depend only on the current robot’s input and in which the robot does not store any internal information regarding previously experienced sensory states. This is achieved by offloading the critical information into the environment (or more precisely into the robot/environmental relationship).

In fact, the behavioral analysis of the best (S) robot indicates that the experienced signals are used to systematically alter the positions assumed by the robot at the end of the central corridor (Fig 4). These positions influence the type of stimuli the robot experiences at the first junction which, in turn, determine whether the robot will turn left or right at the junction. The position of the robot at the end of the first corridor also influences how the turning is realized, i.e., whether the robot produces a tight turn or a wider one, and consequently the position assumed by the robot in the second corridor. Indeed, after experiencing the right-right signals the robot assumes the right most position at the end of the first corridor and then a right position in the second corridor. By contrast, after experiencing the right-left signals the robot assumes the central position at the end of the first corridor and then a left position in the second corridor. This ability to differentiate the relative position assumed in the second corridor on the basis of the position assumed at the end of the first corridor enables the robots to turn in the appropriate direction also at the second junction. The same things happens when the robots travel towards the other two left destinations.

**Fig 5:**
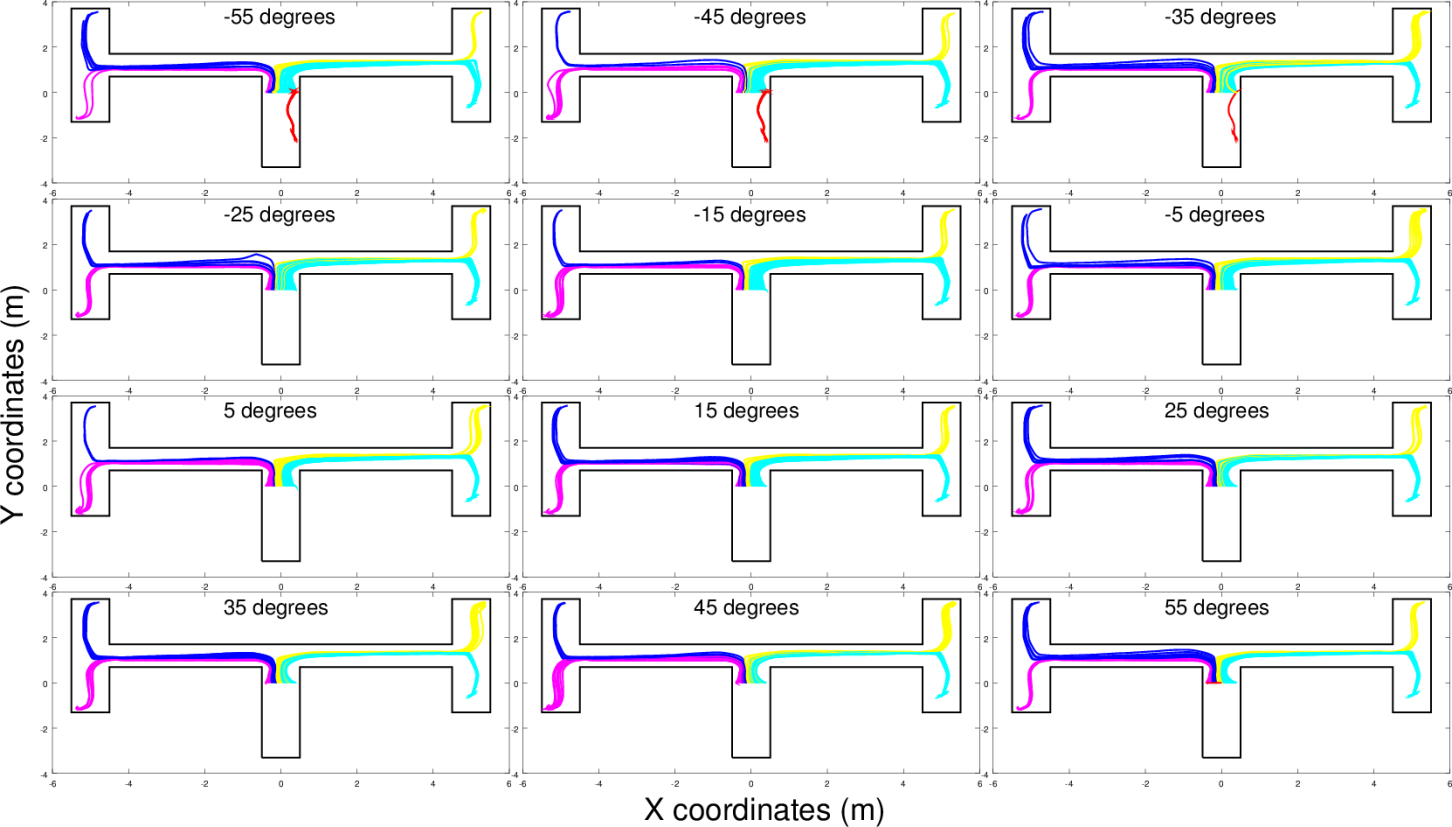
Behavior displayed by the best (S) robot placed initially at the end of the first corridor in different positions (along the x-axis) and orientations. The color of the trajectories corresponds to the destination reached (magenta: left-bottom, blue: left-top, yellow: right-top, and cyan: right-bottom). Trajectories that did not enable the robot to reach any target destination are shown in red.

This interpretation is confirmed by the result of an analysis in which the robot was initially placed at the end of the first corridor with an orientation that systematically varied in the range of [-55, 55]° with respect to the orientation of the first corridor and a position that systematically varied along the x-axis between [-0.4, 0.4]m with respect to the center of the corridor. As shown in Fig 5, in fact, the destination reached by the robot depended primarily on the relative position along the x-axis and secondarily on the orientation of the robot at the end of the first corridor. This means that the robot offloads the information encoding the destination to be reached in its position and orientation. The ineffective behaviors shown in red, which are produced when the robot is initially located near the right wall and oriented toward the right, occur only occasionally in normal conditions (Fig 4) because the robot rarely reaches these positions/orientations when it starts from the beginning of the central corridor.

The fact that the destination reached by the robot depended on the position and the orientation of the robot at end of the first corridor is demonstrated by the fact that the destination the robot will reach at the end of the trial can be predicted with 84% accuracy on the basis of the position and the orientation the robot assumes in the first corridor, 40cm before the first junction. This success rate was obtained by training a feed-forward neural network through a backpropagation algorithm based on a cross-entropy error function [39]. The network included two inputs neurons that encoded the x-position and the orientation of the robot, six hidden neurons and four winner-take-all outputs neurons that encoded the four corresponding destinations. The training set consisted of 6,000 position and orientation vectors and 6,000 corresponding destination vectors. The fact that the target destination could not be predicted in all cases can be explained by considering the effects of noise on sensors and motors. Further evidence demonstrating that the target destination reached by the best (S) and (C) robots depended on the position and the orientation assumed by the robots from the end of the first corridor on are reported below.

**Fig 6:**
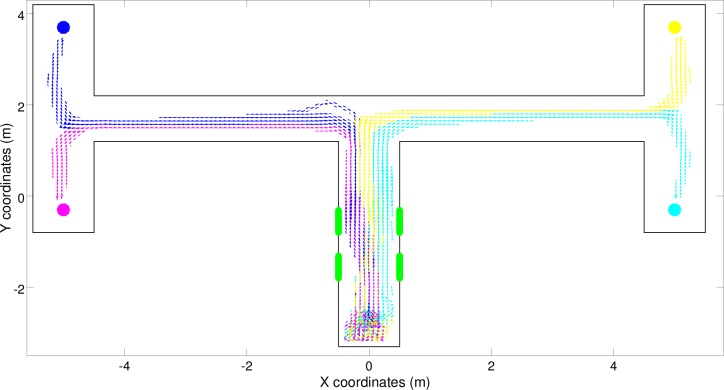
2D vector field displaying the velocity of the robot in varying positions of the x/y plane for the (S) robot. For the sake of clarity, the vectors are shown only for the positions and orientations reached by the robot in natural circumstances (i.e. the positions and orientation assumed by the robot in 300 trials). The multiple arrows displayed in each 75x75mm position cell indicate how the direction and the magnitude of the velocity vector varies as a function of the different orientations assumed by the robot in the corresponding position.

The four behaviors displayed by this robot (indicated by the trajectories shown in magenta, blue, cyan and yellow in Fig 4) are dynamical processes that arise from the robot/environmental interactions and that converge toward four fixed-point attractors. This can be appreciated by observing the four corresponding basins of attraction in the 2D projection of the phase portrait (Fig 6). These basins of attraction enable the robot to reach four different destinations without varying the way it responds to perceived stimuli (i.e. by using a reactive controller that always responds in the same way to the same stimuli independently from the stimuli experienced before). This can be explained by considering that the way in which the robot reacts to perceptual stimuli and the way in which perceptual stimuli change (as a function of the action performed by the robot and of the characteristics of the local portion of the environment) ensure that the robot keeps moving towards the current destination while remaining in the current basin of attraction. To solve the problem, therefore, the robot only needs to enter into the appropriate basin of attraction in the first corridor while it perceives the green stimuli.

**Fig 7:**
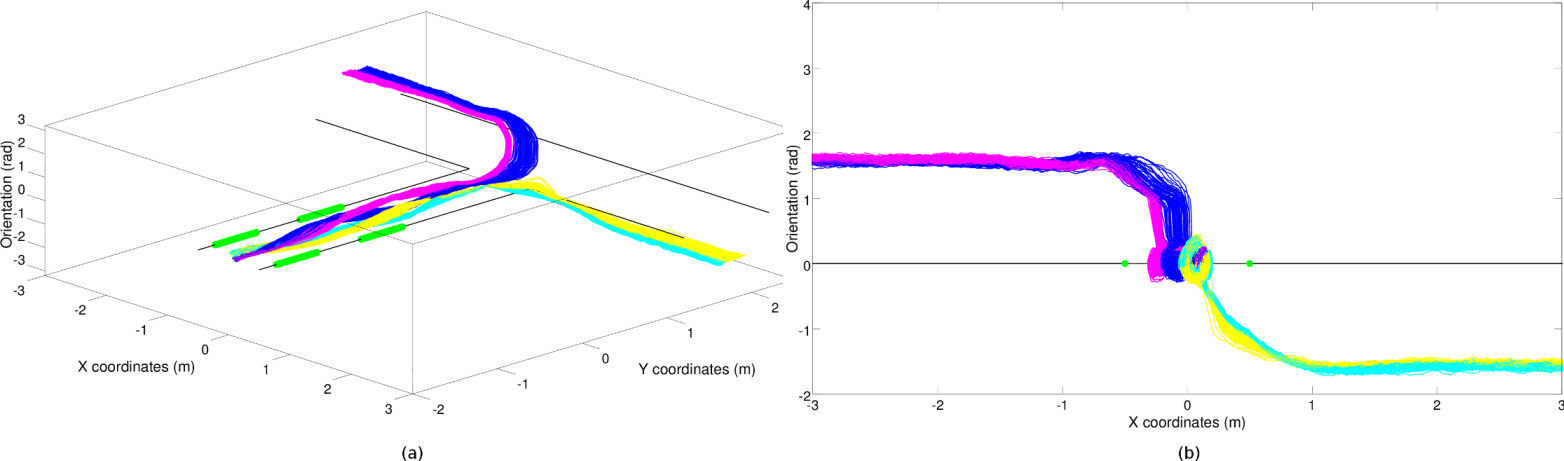
Phase portrait of the robot/environmental dynamics in the case of the best (CR) robot. Data collected during in 300 trials. The vertical axis represents the orientation of the robot in the range [-90, 90]° with respect to the direction of the first corridor. For the sake of clarity the plots refer only to the first-junction portion of the environment. (a) displays a view from which variation on the three dimensions can be appreciated. Instead, (b) shows a 2D orthogonal projection in which one can appreciate only variations along the vertical and horizontal x-axis that correspond to the orientation of the robot and to the position of the robot along the x-axis, respectively.

The basins of attraction also ensure that the behavior of the robot is robust with respect to perturbations caused by noise and environmental variations (within limits). This is because typically small alterations in the robot’s position and orientation do not cause a switch from one basin of attraction to another and consequently do not alter the robot’s destination. Moreover this is also because the effects of small alterations tend to be automatically compensated over time by the convergent nature of the attractors that drive the robot away from the borders that separate the different basins of attraction. Note that, as shown in Fig 7, the state space includes three dimensions (x position, y position and orientation). Consequently, the four basins of attraction are separated also in areas in which they seem to overlap from the perspective of the 2D projection shown in Fig 6.

Selection of the appropriate behavior (i.e. the convergence toward the appropriate basin of attraction) is the result of the bifurcation process that occurs in the first corridor and that is regulated by perception of the green stimuli. In other words, it is the result of the fact that while the robot travels along the first corridor, it varies its position and orientation on the basis of the perceived green stimuli in a way that ensures that at the end of the first corridor the robot assumes a position and orientation that enable it to enter in the right basin of attraction.

For example, let’s consider the trials in which the robots experience the right-right signal (Fig 4, cyan trajectories). In these cases, the robot reacts to the two green stimuli located on the right by moving toward the right side of the corridor. This enables the robot to turn right at the first junction, because it turns right when it encounters a wall ahead and a wall on its right side that is nearer than the wall on its left side, and then to assume a specific position and orientation in the second corridor that enable it to turn right also at the second junction. Thus, the proper movements produced in response to the stimuli perceived in the first corridor ensure that the robot environmental/dynamics will enter into the basin of attraction of the right-right behavior that then guide the robot toward the appropriate destination. During the trials in which the robot experiences the right-left signal, instead, the robot reacts to the signals by moving first right and then left so as to assume a central position within the first corridor (Fig 4, yellow trajectories). This makes the robot turn right at the first junction also in this case. However, the right turn initiated from this position and orientation, drives the robot toward the left side of the second corridor. This in turn enables the robot to then turn left at the second junction. In other words, the position/orientation with which the robot approaches the first junction influences not only whether it turns right or left but also the position/orientation that it takes after the turn in the second corridor, which finally determines whether the robot will turn left or right at the second junction.

There are two reasons for the errors produced by the robot (Fig 4, dashed lines). The first is that in some cases the bifurcation process fails, i.e., the robot is unable to react to the stimuli experienced and, thus, assume the appropriate positions/orientations at the end of the first corridor. Consequently, the robot/environmental system enters into the wrong basin of attraction. This problem particularly affects some of the trials in which the robot experiences the left-right signals. Indeed, in 10.7% of these trials the best (S) robot erroneously navigates toward the right-left destination (Fig 4, dashed lines). This problem occurs when the robot starts from certain specific positions and orientations and/or as a result of noise or when the robot occasionally exits from the right basin of attraction and enters into another, wrong basin of attraction. This typically occurs as a result of noise in areas in which the divergence between two nearby basins of attraction is weak. In the case of the best (S) robot, this second type of problem occurs particularly in the left corridor. Indeed, in this phase the robots traveling toward the left-right destination erroneously enter into the behavioral attractor of the left-left destination in 46.7% of the left-right trials (Fig 4, dashed lines).

**Fig 8:**
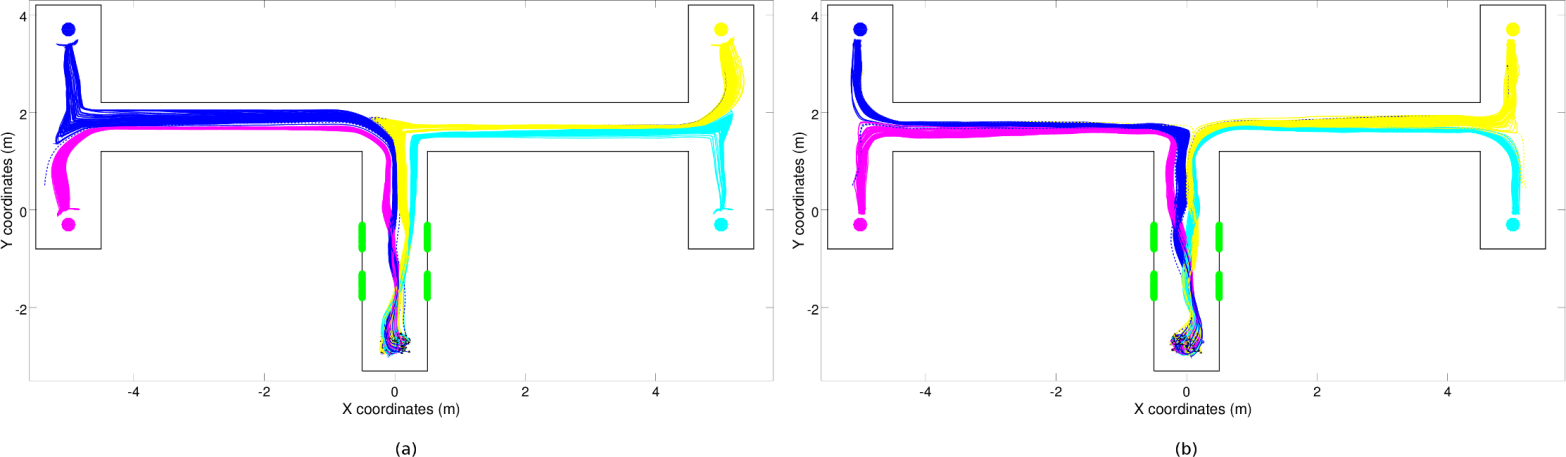
Trajectories produced by the best (C) and (CR) robots in 300 trials, (a) and (b) pictures respectively. Full and dashed lines indicate successful and unsuccessful trials, respectively. The color indicates the corresponding target destination (magenta: left-bottom; blue: left-top, cyan: right-bottom, and yellow: right-top).

**Fig 9:**
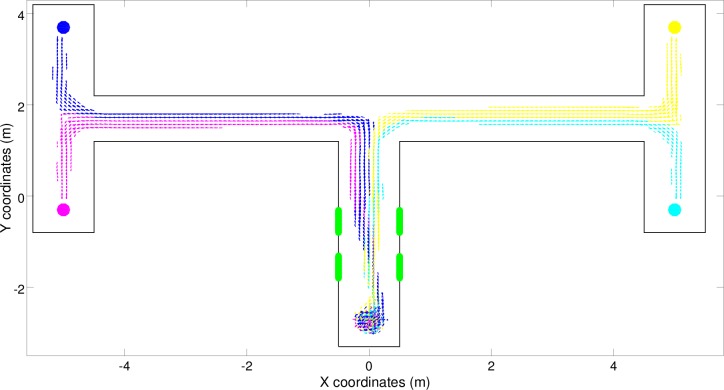
Vector field displaying the velocity of the robot in varying positions of the x/y plane for the (CR) robot. For the sake of clarity, the vectors are shown only for the positions and orientations reached by the robot in natural circumstances (i.e. the positions and orientations assumed by the robot in 300 trials). The multiple arrows displayed in each 75x75mm position cell indicate how the direction and the magnitude of the velocity vector varies as a function of the different orientations assumed by the robot in the corresponding cell and/or as a function of the robot’s internal states.

The behaviors displayed by the best (C) and (CR) robots are qualitatively similar to those displayed by the best (S) robot, see Fig 8. Indeed, also the robot/environmental dynamic of these robots is characterized by four fixed-point attractors (Fig 9). Moreover, the trajectories of these robots also bifurcate in the first corridor to ensure that the robot/environmental dynamic enters into the appropriate basin of attraction.

However, these robots make far fewer errors during the bifurcation phase than the (S) robot thanks to their ability to converge toward similar positions and orientations while they move along the first part of the first corridor (see Fig 8). Indeed, the variability along the x-axis of the positions assumed by (C) and (CR) robots one meter before the green stimuli is significantly lower than the variability of positions assumed by (S) robots (F-Test F(299,299) = 3.114, p<0.001 and F(299,299) = 2.845, p<0.001, respectively). By assuming a relatively un-variant position and orientation before they perceive the green stimuli, (C) and (CR) robots manage to achieve a more reliable bifurcation process than (S) robots.

Also, the errors that occur when the robots exit from their current basin of attraction and enter into another basin of attraction are reduced in (C) and (CR) robots. This is achieved through the synthesis of attractor basins, which produces a greater separation among the trajectories targeted toward different destinations that are produced by (C) and (CR) robots (Fig 8) than among the trajectories produced by the (S) robot (Fig 4).

**Fig 10:**
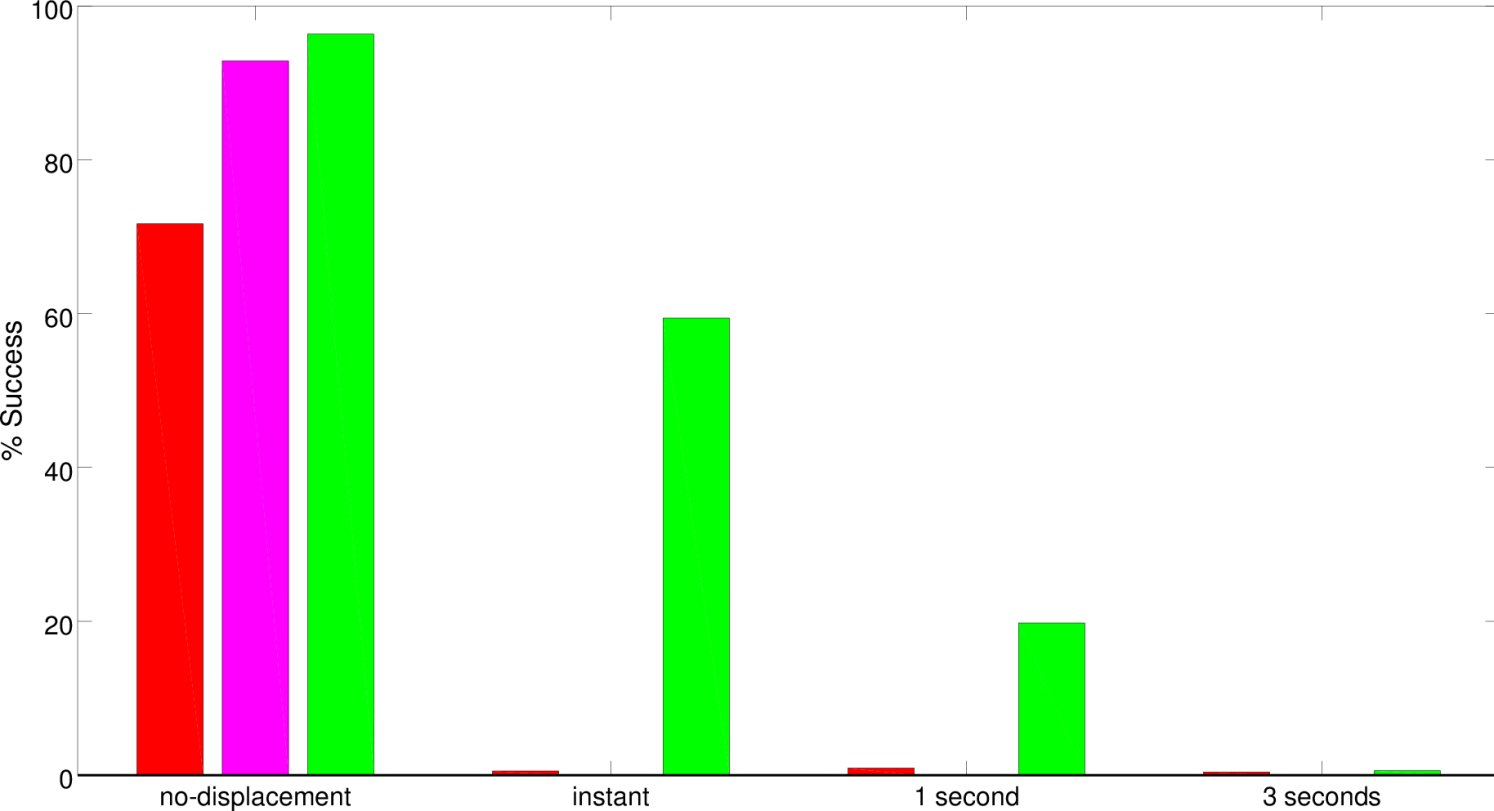
Performance displayed by the best (S), (C), and (CR) robots during a post-evaluation test in which they were randomly displaced from their current basin of attraction into another one. The red, magenta and green bars represent the performances of the best (S), (C) and (CR) robots, respectively. Each bar represents the percentage of trials in which the robots were able to reach the appropriate destination despite the displacement. The “instant” condition refers to a situation in which immediately after the displacement the robots were allowed to move normally. The “1 second” and “3 seconds” conditions refer to a situation in which the robots were unable to move for 1 and 3 seconds, respectively. The “no-displacement” condition refers to a normal situation in which the robots were not subjected to displacements. During each trial the robot was displaced when it reached an imaginary line located 40cm before the first junction, 40cm after the first junction, in the middle of the second corridor or 40cm before the second junction. Data were collected from 2400 trials.

Finally, in some cases the best (CR) robot also displayed the ability to re-enter into the basin of attraction in which it was previously located when it erroneously moved to another basin of attraction. This enables the robot to recover from some of the errors of this type caused by noise. This is demonstrated by the results collected during a post-evaluation test in which the robot was systematically displaced from its current basin of attraction to another one. The basin of attraction in which the robot is displaced is chosen randomly from among the other available ones, i.e., from the other three alternative basins of attraction before the first T-junction or between the only alternative attractor after the first T-Junction. The post-evaluation test was repeated in three conditions in which the robot was allowed to move normally and in which it was blocked for 1 or 3 seconds after displacement. Analysis of the results indicates that the best (CR) robot was able to recover from this type of displacement in 60% of cases in which it was allowed to move normally after the displacement and in 20% of cases in which it was blocked in the displaced position and orientation for 1 second (Fig 10). The best (S) and (C) robots, instead, were able to recover from displacements only in a negligible percentage of cases (Fig 10). None of the robots were able to recover from displacements after being blocked in the displaced position and orientation for 3 seconds.

Overall the data reported in this section indicate that all robots offloaded information concerning the stimuli they experienced in the position and orientation they assumed from the end of the first corridor on and used this information to move toward the appropriate destination and to preserve the relevant information (i.e. to maintain a specific relative position and orientation with respect to the environment). (C) and (CR) robots also exploited the possibility of integrating sensory-motor information over time to regulate their motor behavior in the very first portion of the first corridor so as to reduce the variability with which they reached the green stimuli. Moreover, (C) and (CR) robots also exploited the possibility of integrating sensory-motor information over time to partially filter out the effect of noise affecting their sensors and motors and to better separate the trajectories produced while they navigated toward different target destinations.

**Fig 11:**
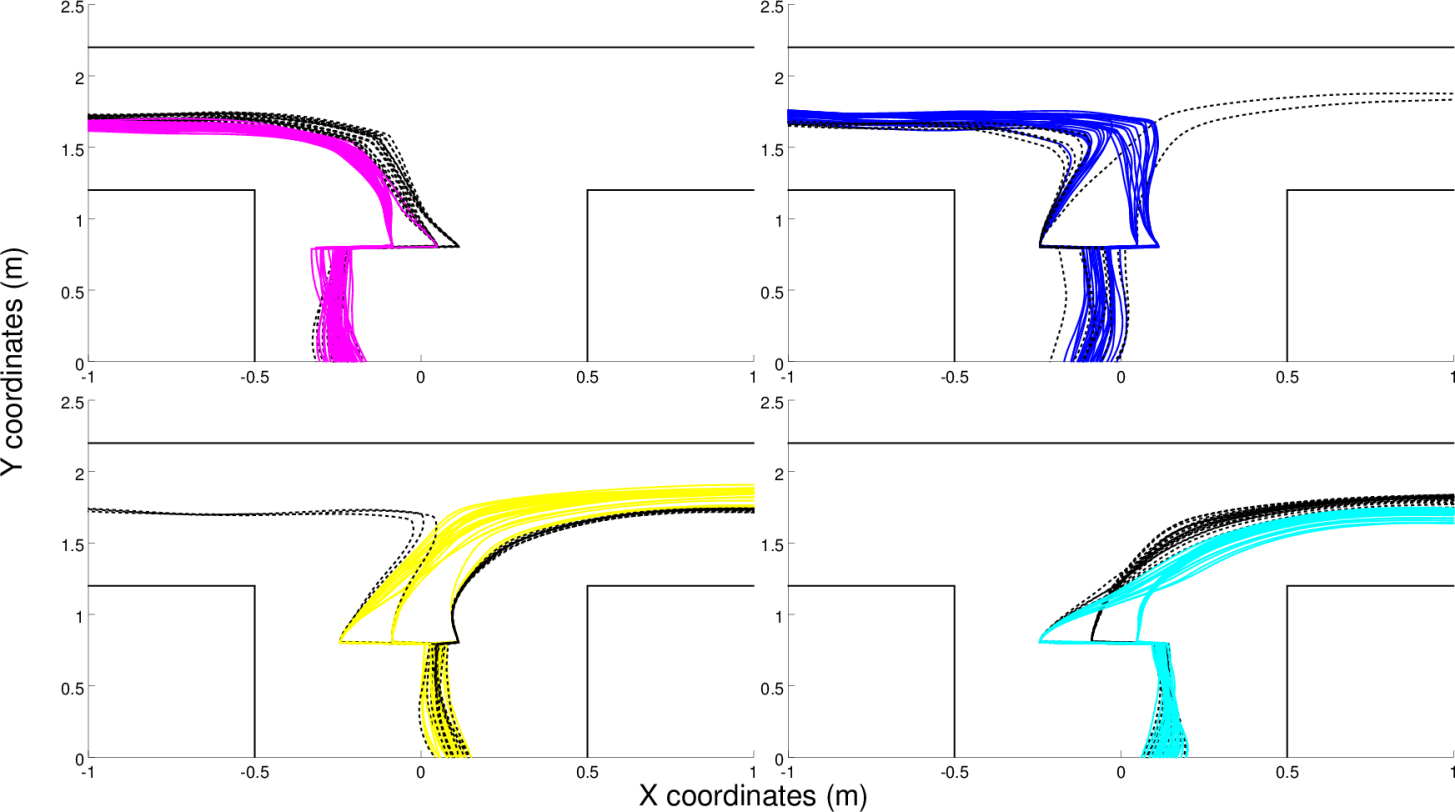
Trajectories displayed by the best (CR) robot at the first T-junction during a post-evaluation test in which the robots were displaced into one position and orientation located within one of the other three basins of attraction. The displacement was performed when the robot reached a distance of 40cm from the first junction. After the displacement the robot was allowed to move immediately (i.e. it was not blocked). Data were collected from over 200 trials, 50 for each combination of green stimuli. The colors indicate the target destinations (magenta: left-bottom, blue: left-top, cyan: right-bottom, and yellow: right-top). The black trajectories indicate the trials in which the robot reached a wrong destination, i.e. was unable to re-enter the correct basin of attraction after the displacement.

The destination reached by (S) and (C) robots was determined by the position and the orientation assumed by the robots from the end of the first corridor on and was not affected by the type of stimuli experienced previously. Indeed when these robots were displaced from their current position inside a certain basin of attraction into a position and an orientation located in a different basin of attraction, they navigated towards the target destination corresponding to the second basin of attraction in 98% of cases. The destination reached by (CR) robots, instead, also depended on the state of the internal neurons that encode information about previously experienced sensory states. Indeed, displaced (CR) robots navigated toward the destination corresponding to the basin of attraction in which they were located before the displacement in 60% of cases. They managed to compensate the effect of the displacement by re-entering the basin of attractions in which they had been previously located. They navigated toward wrong destinations only in the remaining 40% of cases (see Fig 11).

### Limiting cognitive offloading does not promote but rather prevents the synthesis of effective solutions

Here we report a series of experiments carried out to verify whether the discovery of sub-optimal reactive strategies that rely on cognitive offloading prevents the discovery of better cognitive solutions. In other words, we verify the hypothesis that cognitive offloading constitutes a sort of shortcut that enables evolving individuals to improve their adaptive ability up to a certain level through the utilization of solutions that are parsimonious from the perspective of the control system of the robot but that prevent the discovery of more complex and effective strategies. To achieve this objective we analyzed the solutions found by evolving robots in situations in which the possibility of relying on cognitive offloading was reduced or prevented.

One way to reduce the possibility of relying on cognitive offloading in the case of our experiments was to drastically reduce the width of the corridors. As we have seen, in fact, robots offload information concerning the type of green stimuli they have experienced by assuming different relative positions/orientations inside corridors. The utilization of narrow corridors severely restricts the possibility of carrying out this type of offloading.

**Fig 12:**
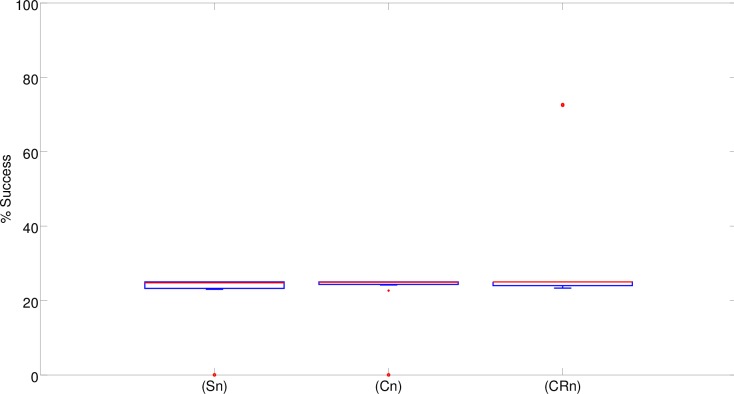
Performances obtained in the control experiment with narrow corridors. The boxplots show the percentage of successful trials carried out by the best 10 robots evolved in the (S), (C), and (CR) experimental conditions in 10 corresponding replications of the experiment. Boxes represent the inter-quartile range of the data. The horizontal lines inside the boxes mark the median values. The whiskers extend to the most extreme data points within 1.5 times the inter-quartile range from the box. Circles mark the outliers.

Analysis of the results obtained in a series of control experiments in which the width of the corridors was set to 29cm only indicates that, as expected, the use of highly constrained environmental conditions prevents the exploitation of cognitive offloading, i.e., the evolution of solutions analogous to that described in the previous section (results not shown). However, analysis of the performance of the robots evolved in this condition indicates that the elimination of solutions based on cognitive offloading does not lead to effective solutions. In fact, it causes a drastic reduction of the robots’ performance with respect to the normal condition (Fig 12). This implies that the elimination of solutions relying on cognitive offloading does not facilitate the evolution of alternative cognitive solutions. In other words, the lack of evolution of effective cognitive solutions cannot be explained simply by the availability of cognitive offloading “shortcuts”.

Indeed, in most of the replications the best (Sn), (Cn) and (CRn) evolved robots were able to navigate correctly to only one of the four destinations and consequently succeeded in about one fourth of the trials. The evolved robots managed to navigate to one of the four correct destinations in most of the trials. Consequently, the impact of the errors that occurred when the robots remained stuck, navigated erroneously back toward the central corridor or crashed into obstacles was marginal. We used (Sn), (Cn) and (CRn) to indicate the robots evolved in the narrow corridor condition. In only two replications, the best (CRn) robots managed to navigate correctly toward three out of the four destinations by succeeding in about three fourths of the trials (Fig 12). The performance of these two robots was similar to that achieved by the best (S) robot (Fisher exact test, p = 0.747 and p = 0.797), which could only rely on reactive strategies (Fig 3). However it was significantly worse than that of the better (C) and (CR) robots, (Fisher exact test, p<0.001). Overall, the performance of the robots that evolved in these control experiments (Fig 12) was significantly worse than the performance obtained in the standard experiments (Fig 3) (Mann-Whitney U, p<0.001 for (S) and (C), and p = 0.01 for (CR)).

Another mechanism that can be used to discourage evolving robots from relying on cognitive offloading is to randomly vary the position and orientation of the robots while they travel in the maze. To investigate the effect of this type of perturbation we carried out a series of control experiments in a standard maze in which every 50ms the position and the orientation of the robot was perturbed with a 15% probability. The perturbations were created by displacing the robot to the left or the right of a distance d and by varying the orientation of the robot by an angle a, where d and a are selected randomly with a uniform distribution within the range [3, 9]cm and [-15, 15]°, respectively. This type of perturbation drastically reduces the utility (usefulness) of offloading information in the relative positions and orientation of the robot in the environment. Once again, the hypothesis under verification is whether the introduction of a constraint that discourages the development of cognitive offloading solutions will favor the development of alternative, and possibly better, solutions. We used (Sp), (Cp) and (CRp) to indicate the robots evolved in the standard maze subjected to position and orientation perturbations during evolution.

**Fig 13:**
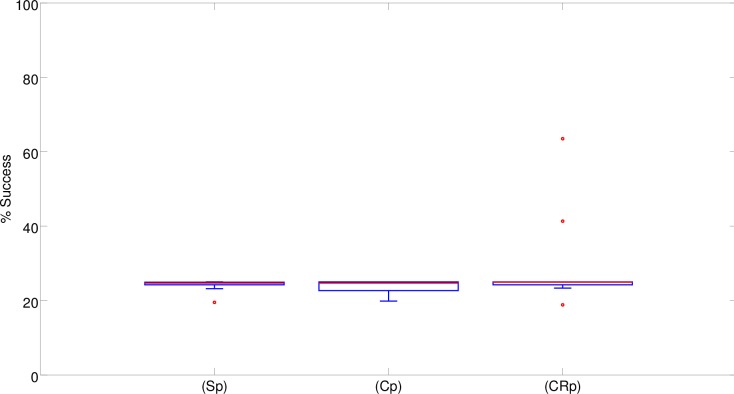
Performance obtained by robots that were subjected to position and orientation perturbations in all trials during 4000 generations. Data were obtained by post-evaluating the robots in a normal condition in which they were not subjected to perturbations. The boxplots show the percentage of successful trials carried out by the best 10 robots evolved in the (Sp), (Cp), and (CRp) experimental conditions in 10 corresponding replications of the experiment. Boxes represent the inter-quartile range of the data. The horizontal lines inside the boxes mark the median values. The whiskers extend to the most extreme data points within 1.5 times the inter-quartile range from the box. Circles mark the outliers.

The fact that this form of perturbation drastically reduces the usefulness of cognitive offloading is demonstrated by the fact that the performance of (Sp) robots, which can only rely on reactive strategies, drops to very low levels (Fig 13). The introduction of perturbations, however, causes a significant drop in performance with respect to the experiments without perturbations also in the case of (Cp) and (CRp) robots (Mann-Whitney U, p<0.001) (Fig 13). Notice that Fig 13 displays the results of a post-evaluation test in which the robots are evaluated in the absence of position and orientation perturbations.

Also in this case, therefore, the introduction of constraints that discourage the development of reactive solutions relying on cognitive offloading does not promote the evolution of effective cognitive solutions but rather prevents the possibility of synthesizing good solutions of any kind.

As we will show in the next section, this negative result is not due to the impossibility of generating solutions that are able to compensate the effect of position and orientation perturbations. Indeed, evolving robots can solve the navigation problem in a close to optimal manner and can neutralize to a good extent the effect of position and orientation perturbations, providing that the constraints which discourage the development of cognitive offloading strategies are not too strong.

### The acquisition of reactive strategies promotes the evolution of cognitive capabilities

As demonstrated in the previous section, preventing or severely limiting the possibility of developing strategies based on cognitive offloading prevents the development of effective solutions. In this section, we demonstrate how the acquisition of reactive strategies promotes the evolution of cognitive solutions that enable the robots to accomplish their task also in conditions that cannot be mastered appropriately only by using cognitive offloading strategies.

**Fig 14:**
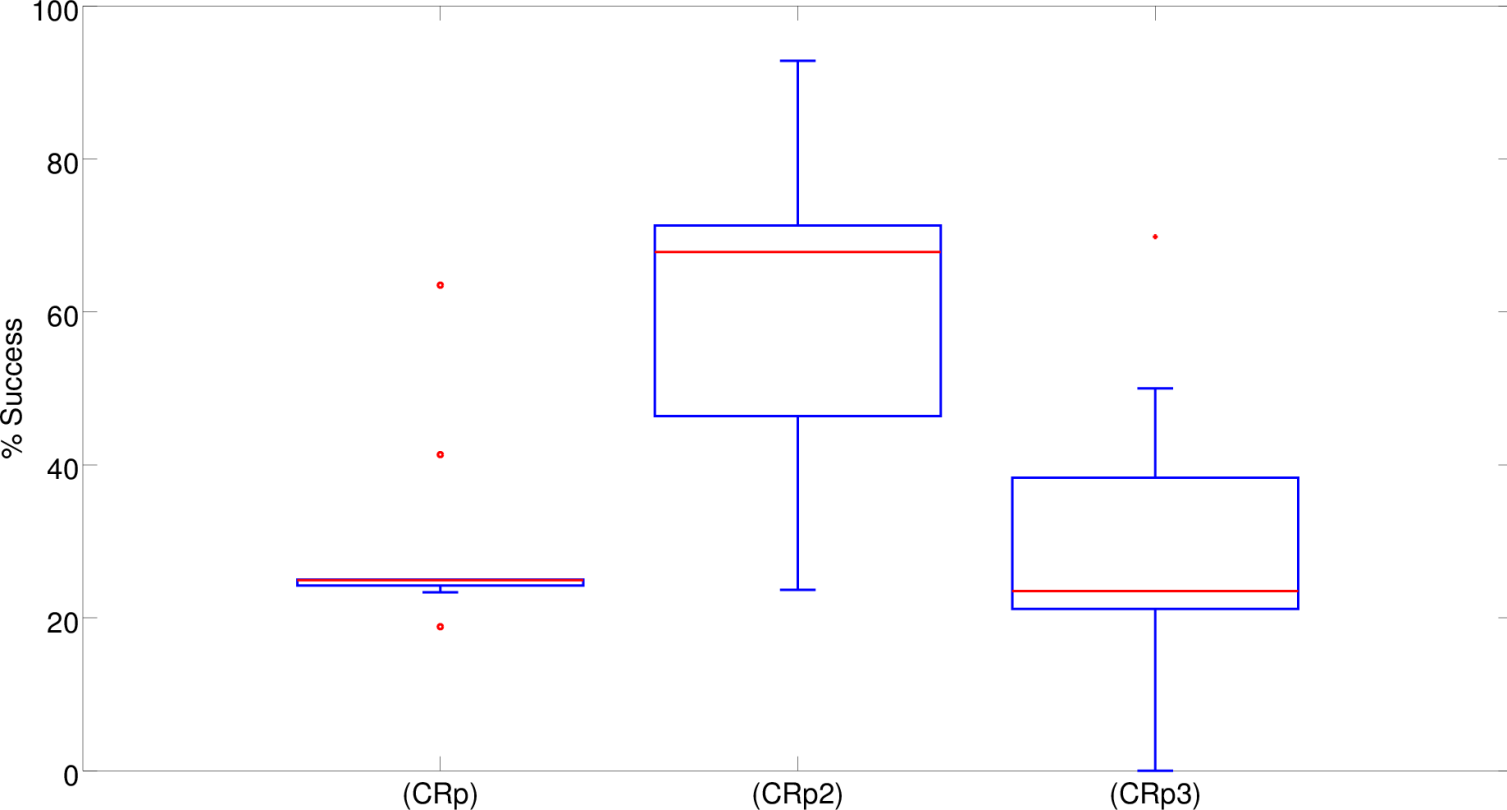
Performances obtained in experiments in which the robots were subjected to position and orientation perturbations in all cases (CRp), during half of the trials (CRp2) and during the second evolutionary phase (CRp3). Data obtained by post-evaluating the robots without position and orientation perturbations. The boxplots show the percentage of successful trials carried out by the best 10 robots evolved in each of the experimental conditions in 10 corresponding replications of the experiment. Boxes represent the inter-quartile range of the data. The horizontal lines inside the boxes mark the median values. The whiskers extend to the most extreme data points within 1.5 times the inter-quartile range from the box. Circles mark the outliers.

To verify this hypothesis we carried out a new series of experiments in which we weakened the constraints that discourage the utilization of cognitive offloading and increase the demand for cognitive solutions. This was carried out by subjecting the robots to position and orientation perturbations in only half of the trials (Fig 14, CRp2 condition) or by subjecting the robots to perturbations only during the second phase of the evolutionary process, i.e., from generation 2001 to 4000 (Fig 14, CRp3 condition). Notice that the Fig 14 displays the results of a post-evaluation test in which the evolved robots were not exposed to position and orientation perturbations.

The performances of the robots that were subjected to perturbations in only half of the trials are significantly better than the performances of the robots that experienced perturbations in all trials (Fig 14, CRp2 and CRp, Mann-Whitney U, p = 0.004). The performances of the robots that were subjected to perturbations during the second phase of the evolutionary process, instead, did not differ significantly from those of the robots that were subjected to perturbations during all generations (Fig 14, CRp3 and CRp, Mann-Whitney U, p = 0.36).

**Fig 15:**
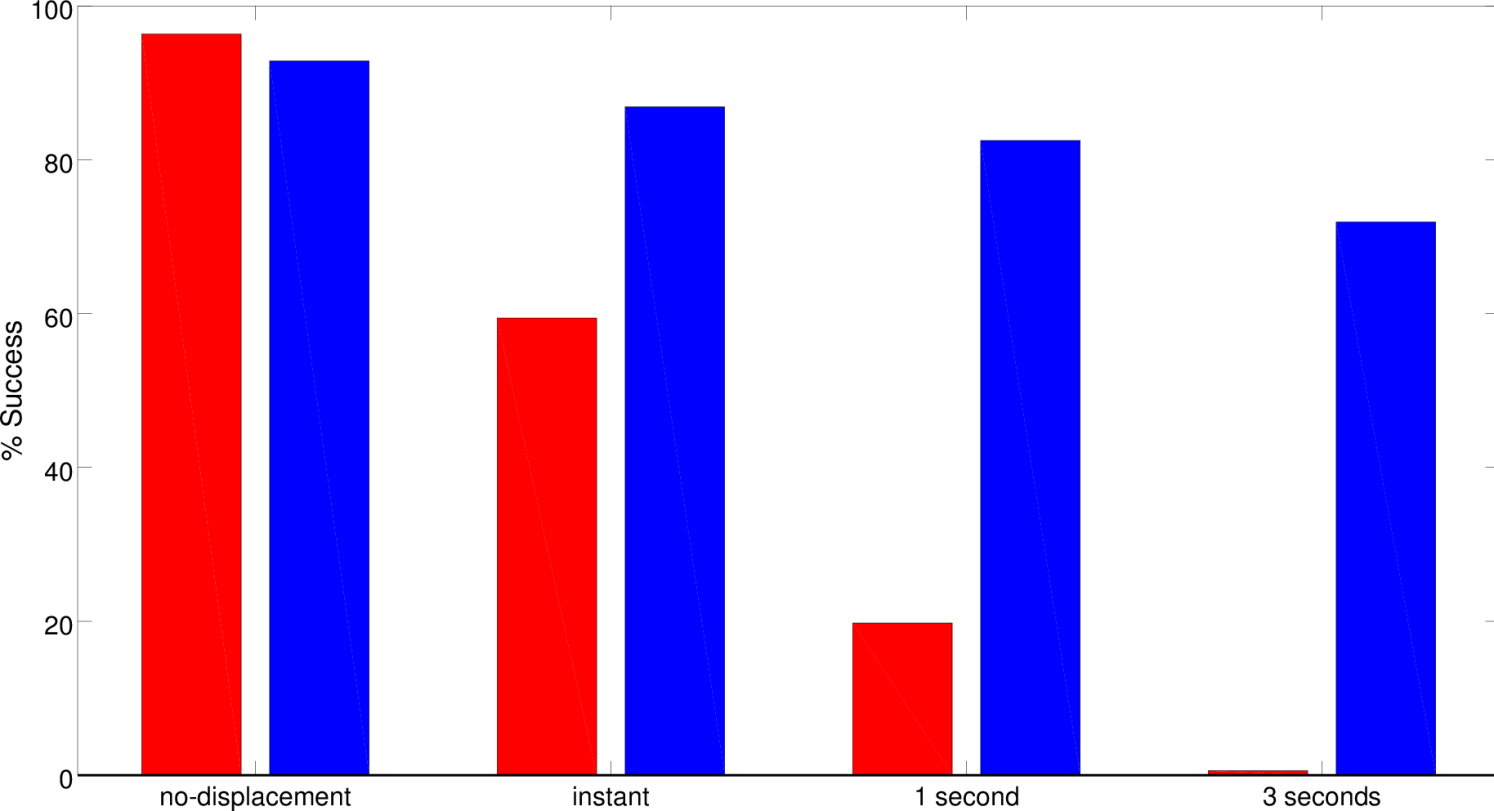
Performance displayed by the best (CR) and (CRp2) robots during a post-evaluation test in which the robots were systematically displaced into a position and orientation located in a different basin of attraction (red and blue bars, respectively). Each bar represents the percentage of trials in which the robots were able to reach the appropriate destination despite the displacement. The “instant” condition refers to a situation in which the robots were allowed to move normally immediately after the displacement. The “1 second” and “3 seconds” conditions refer to situations in which the robots were blocked for 1 and 3 seconds, respectively, after the displacement. The “no-displacement” condition refers to a normal situation in which the robots were not subjected to displacements. The robots were displaced once during each trial. The displacement occurred when they reached an imaginary line located 40cm before the first junction, or 40cm after the first junction, or in the middle of the horizontal corridor, or 40cm before the second junction. Data collected in 2400 trials.

The fact that the robots evolved in the CRp2 condition relied on effective cognitive mechanisms can be demonstrated by post-evaluating the best robot in a control condition in which it was systematically displaced from its current position and orientation into another position and orientation located in a different basin of attraction through the same procedure described in Section 3. As can be seen, unlike the best CR robot, the best CRp2 robot is able to recover from the displacements in most cases, also in the condition in which it is blocked for three seconds after being displaced (Fig 15). The robot compensates for the effect of displacement by re-entering into the basin of attraction in which it was located before the displacement. This is carried out by storing in its internal states information encoding the type of basin of attraction in which it is located and by using this information to re-enter into the previous basin of attraction, as shown in Fig 11. The best CR and CRp2 robots make use of the internal states to neutralize the effects of this type of displacement (see Figs 10 and 15). However, the best CRp2 displays much better ability. This is not surprising, because CRp2 robots were subjected to position and orientation perturbations during evolution. In normal conditions, i.e., without displacements or perturbations, the performances of the best CR and CRp2 controllers do not differ statistically (Fisher Exact Test, p = 0.308).

**Fig 16:**
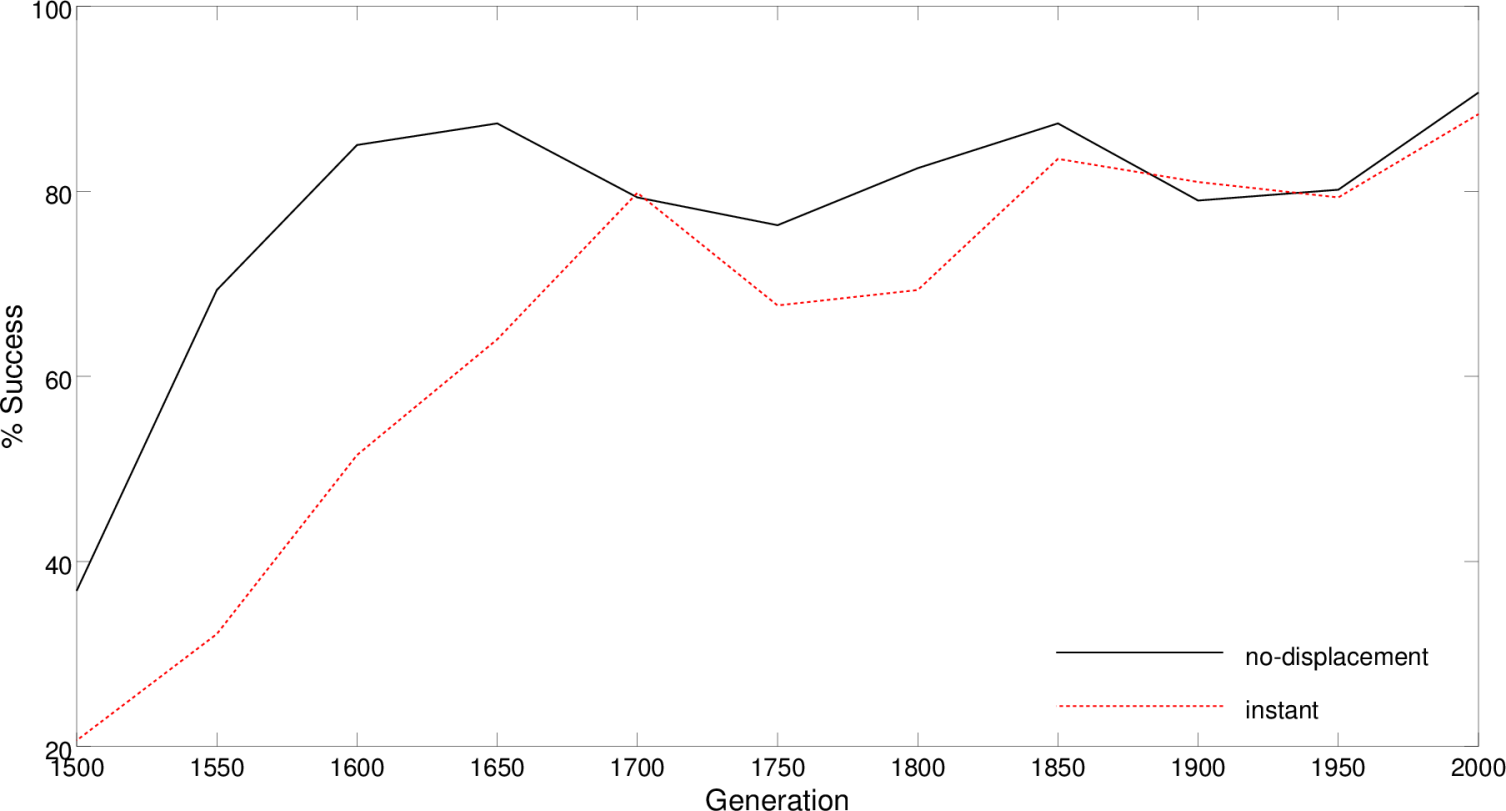
Performance displayed by the best CRp2 during the course of the evolutionary process in a no-displaced and displaced condition (black and red lines, respectively). In the latter condition the robot was allowed to move immediately after the displacement. Data were collected every 50 generations from generation 1500 to 2000 and averaged over 600 trials in the case of the no-displacement condition and 2400 trials in the case of the displaced condition. The displacement occurs when the robot reaches an imaginary line located 40cm before the first junction, or 40cm after the first junction, or in the middle of the horizontal corridor, or 40cm before the second junction.

The fact that the development of cognitive offloading strategies supports the development of effective cognitive abilities, such as those displayed by the best CRp2 robot, is also demonstrated by the analysis of the course of the evolutionary process in the case of the best replication of the experiment. In fact, the post-evaluation of the best robots in every 50 generations indicates that the development of the ability to master the trials not affected by displacements precedes the development of the ability to also master the trials subjected to displacement (Fig 16). The performances in the two conditions differed significantly from generation 1500 to 1800 with the exception of generation 1700 (Fisher Exact Test, p<0.05). We focused on this 500 generations period because in the case of the best replication this is the phase in which most progress occurs. Thus, the development of a strategy that operates primarily on the basis of cognitive offloading and that enables the robots to handle the navigation task in the normal condition but not in the condition with displacements supports the development of a hybrid strategy that relies also on internal states and that enables the robots to also master the trials affected by displacements.

Incidentally, the internal neurons of the (CR) and (CRp2) robots are somewhat similar to the hippocampal cells of rodents located in maze environments that encode information about the location of the animal in the maze and about the trajectory the animal is performing and will perform to reach the target destination [40–42]. However, a detailed analysis of the relation with these neurophysiological findings is outside the scope of this paper.

At this point we will try to explain why evolving robots: (i) always develop cognitive offloading strategies, (ii) are unable to develop effective strategies relying exclusively on internal states even in conditions in which the possibility of using cognitive offloading is completely ruled out, (iii) are able to extract internal states encoding target destinations that are maintained over time and are used to compensate the effects of position and orientation perturbations.

We believe that to explain these results we have to appreciate the full complexity of the task. In particular, we must consider that the problem involves both the ability to travel in the maze environment by avoiding collisions and inversions in the direction of motion and the ability to turn in the appropriate direction at junction areas. The most straightforward way of navigating in the maze and avoiding collisions is to use a reactive strategy that regulates the direction of the robot on the basis of the current state of the infrared sensors. In other words, the ability to navigate in the maze environment necessarily requires use of a reactive strategy. The capacity to turn in the appropriate direction at junction areas, instead, can be obtained either by exploiting primarily external cues generated through cognitive offloading or by exploiting primarily internal cues generated by integrating sensory-motor information over time. Using a mixed strategy that operates both on the basis of reactive rules for the purpose of navigation and on the basis of more complex rules for the purpose of decision-making at junctions necessarily requires the incorporation of mechanisms that sort out the conflicts arising between reactive and non-reactive control rules.

As an example of conflict, let’s consider the case of a robot that decides which direction to take at a junctions on the basis of its internal state. Moreover, let’s imagine that the robot is located at the beginning of the first junction and that is traveling toward one of the two left destinations. During the first and the third part of the junction negotiation the robot should not turn too sharply left to avoid colliding with the left walls. During the central part of the junction negotiation, instead, the robot should definitely turn left following the indication coming from its internal state. The conflict arising between obstacle avoidance and the decision-making rules that control the direction of turns at junctions could be solved by regulating the relative importance of the two rules on the basis of the relative position of the robot within the junction. However, the robot does not have access to position information. It only perceives the proximity of nearby obstacles. This implies that the robot would be forced to determine the relative importance of the two alternative control rules on the basis of the indirect, noisy, and incomplete information provided by its sensors. Consequently, this implies that the probability that the robot will fail as a result of ineffective regulation is not negligible.

Instead, the behavioral attractor strategies displayed by evolved robots do not require differentiating the way in which the robots react to sensory states experienced in different portions of the environment. Indeed, once the robots enter into the right basin of attraction, they just need to keep moving on the basis of simple reactive rules in both corridors and junctions. One reason why evolving robots always select cognitive offloading solutions is that these strategies are more robust, less prone to errors with respect to strategies in which turning decisions are made on the basis of internal states.

The second reason why evolution always converges toward cognitive offloading strategies is that preparatory actions that anticipate in part the execution of the required behavior are adaptive and therefore tend to be selected. This implies that the individuals that anticipate the movement toward the left or the right side of the corridor during the trials in which they should turn toward that side at the first and/or at the second junctions tend to be selected. This produces a progressive anticipation of the time when the turning actions are initiated that ultimately leads to a situation in which they are initiated up to the point when the robot perceives the green stimuli. This in turn eliminates the need to extract and use internal states that encode information about previously experienced stimuli. Indeed, we might say that in the evolved robots the left or right turning behaviors produced at the first and second junctions are initiated already during the first half of the first corridor when the robot perceives the position of the green stimuli. In other words, by anticipating action execution through preparatory actions the robots manage to transform a time delay task into a simpler problem that does not include any temporal offset between the perception of stimuli and the initiation of the action afforded by the stimuli. Overall this implies that the selection of cognitive offloading strategies of this type is inevitable, at least in the case of the double T-Maze experimental setting.

The tendency to anticipate behaviors through the execution of preparatory actions provides two advantages: it enables the execution of smoother transitions between behaviors (i.e. between the navigation behavior performed within the corridors and the turning behavior performed within the junctions), and it enables reducing and/or eliminating the time delay between the moment when the stimuli affording a given behavior are experienced and the moment when the behavior afforded by the stimuli is executed.

As we have showed above, however, the ability to solve the time-delay problem through preparatory actions does not necessarily prevent development of the ability to extract information encoding the type of basin of attraction in which the robot is currently located or the green stimuli that the robot experienced and the development of an ability to use these internal states to navigate to the appropriate destination. Indeed, as we have seen, (CR) robots display the ability to re-enter into the basin of attraction in which they were previously located after being displaced into another wrong basin of attraction. In the case of robots that are subjected to a moderate level of position and orientation perturbation (CRp2), this cognitive capability is so effective that it enables the robots to compensate for the effect of the displacement in most cases, even when the robots are blocked for three seconds after the displacement. This type of redundant solution enable these robots to exploit the advantage of preparatory actions and to avoid the problems caused by noise and by position perturbations.

Anticipation is a widespread phenomena in sequential motor control. The preparatory actions that support the realization of effective grasping behaviors are an example of this. These preparatory actions involve appropriate modification of the posture of the hand performed during execution of the reaching behavior that precedes the grasping action [43]. A second example is constituted by the co-articulatory movements produced by sign language interpreters engaged in fingerspelling. In fact, the posture of the hand that is used to indicate a letter is influenced by the posture that the hand should assume later to indicate the following letters [44].

## Discussion

In this paper we investigated whether the development of reactive solutions promotes or prevents the development of cognitive solutions to problems in which reactive control policies enable the achievement of sub-optimal performance only. For this purpose we carried out a series of experiments in which evolving robots had to solve a time-delay task in a double T-maze environment in which the destination to be reached depended on the stimuli perceived by the robot during the initial phase of the navigation. The problem chosen is qualitatively similar but more complex than the tasks used in previous studies that investigated the evolution of cognitive capabilities [19][27–31]. The additional complexity is that our task involves a greater number of different destinations, requires making two subsequent stimuli-dependent decisions, involves a longer time delay between the moment in which the robot experiences the stimuli and the moment in which it should make the corresponding turning decisions, and involves variations affecting the initial position and orientation of the robot, the position of the stimuli and the size of the environment.

Analysis of the experiment in which the robots were provided with reactive controllers confirmed that the problem does not allow for optimal, or close-to-optimal, reactive solutions (Fig 3). Surprisingly, however, reactive robots managed to solve the task to a large extent. Analysis of the solutions discovered by the evolving robots indicate that this is achieved by exploiting cognitive offloading. Indeed, the evolved robots display an ability to extract critical states, store these states in the robot/environmental relations and regulate their behavior on the basis of their relative position in the environment (Fig 4).

Analysis of the robots provided with richer neural controllers indicated that the possibility of storing internal states enables the evolving robots to achieve close-to-optimal performance, i.e., to achieve better performance with respect to reactive robots (Fig 3). Analysis of these experiments indicates that cognitive offloading also plays a key role in these robots (Figs 8 and 10). The achievement of better results is due to the development of additional cognitive capabilities that overcome the limitations displayed by robots that operate on the basis of reactive controllers only. The results obtained thus indicate that the development of the reactive strategies based on cognitive offloading does not prevent the development of solutions that rely on cognitive capabilities.

This conclusion is further supported by results obtained in other experiments in which the usefulness of cognitive offloading was reduced or eliminated by using an environment formed by narrow corridors or by subjecting evolving robots to position and orientation perturbations. As expected, the robots evolved in these conditions relied less or not at all on cognitive offloading. This, however, did not enable the robots to discover alternative strategies for solving the task. This simply led to the robots displaying rather ineffective solutions (Figs 12 and 13). Overall, this indicates that the elimination of cognitive offloading does not promote but rather prevents the synthesis of effective solutions.

Finally, we demonstrated how the acquisition of reactive strategies promotes the evolution of cognitive strategies, or better of hybrid strategies that include both cognitive offloading and cognitive mechanisms. These hybrid strategies enable the robots to navigate toward the appropriate destination also after being displaced into another basin of attraction and also after being blocked there for three seconds (Figs 14 and 15). This type of solution was obtained by weakening the constraints that reduce the usefulness of cognitive offloading (i.e. by perturbing the position and orientation of the robot in only half of the trials). This, in fact, creates the appropriate demand for the development of cognitive abilities without preventing the development of cognitive offloading strategies.

Overall these results indicate that reactive strategies relying on cognitive offloading do not necessarily constitute a dead end that might retard or prevent the evolution of better cognitive strategies. On the contrary, they constitute an important component of effective solutions and can co-exist and support the development of complementary cognitive capabilities. For results collected in human subjects that indicate how cognitive offloading can favor the acquisition of abstract concepts see [45].

The importance of the incremental nature of the evolutionary process and of the acquisition of reactive strategies in the synthesis of better cognitive strategies is further demonstrated by the analysis of the course of the evolutionary process. Indeed, the evolution of the cognitive offloading ability precedes the evolution of the cognitive capabilities that use internal states to determine the travel destination (Fig 16).

One question that remains open is whether solutions that are purely cognitive, i.e., that do not also rely on cognitive offloading, exist and can be discovered. We believe that the existence of this type of solution cannot be taken for granted, at least in the context of the domain considered in this paper.

In general, analysis of the characteristics of evolved strategies suggests that reactive and cognitive components of control policies should not be considered as neatly separable entities performing well-differentiated functions. Regulation of the robot’s actions performed on the basis of internal states should always be integrated with regulation of the robot’s actions, which is carried out on the basis of currently perceived states. In the context of our experiments this implies that the efficacy of a cognitive component of the robot policy that determines the turning direction at T-junctions on the basis of internal states strongly depends on the way the robot reacts to perceived stimuli independently of the value of its internal states, and vice versa. Moreover, use of a cognitive offloading strategy for the purpose of storing information about previously experienced stimuli does not necessarily conflict with use of internal states that have the same function. On the contrary, the combined use of two alternative mechanisms with different characteristics for achieving the same function might provide advantages. If we embrace a less simplified view of the relation between reactive and cognitive components we see fewer reasons to expect interferences and more reasons to expect synergies, like those we found in our experiments.
